# The Long-Term Effect of Cochlear Implantation on Tinnitus: A Systematic Review and Meta-Analysis

**DOI:** 10.3390/diagnostics14182028

**Published:** 2024-09-13

**Authors:** Yutian Li, Huiwen Yang, Xun Niu, Yu Sun

**Affiliations:** 1 Department of Otorhinolaryngology, Union Hospital, Tongji Medical College, Huazhong University of Science and Technology, Wuhan 430022, China; 2 Institute of Otorhinolaryngology, Union Hospital, Tongji Medical College, Huazhong University of Science and Technology, Wuhan 430022, China; 3 Hubei Province Clinic Research Center for Deafness and Vertigo, Wuhan 430022, China; 4 Hubei Province Key Laboratory of Oral and Maxillofacial Development and Regeneration, Wuhan 430022, China

**Keywords:** tinnitus, cochlear implantation, CI, curation, meta-analysis

## Abstract

Objective: This systematic review investigates the long-term effect of cochlear implantation (CI) on clinical outcomes in tinnitus patients with sensorineural hearing loss (SNHL). Database Sources: PubMed, Embase, and the Cochrane Library were searched from inception to 30 April 2024. Manual searches of reference lists supplemented these searches when necessary. Review Methods: Original studies included in the meta-analysis had to contain comparative pre- and postoperative data for SNHL patients who underwent CI. Outcomes measured were the Tinnitus Handicap Inventory (THI), Visual Analog Scale (VAS), and Tinnitus Questionnaire (TQ). Results: A total of 28 studies comprising 853 patients showed significant tinnitus improvement after CI: THI mean difference (MD) −14.02 [95%CI −15.29 to −12.76, *p* < 0.001], TQ MD −15.85 [95%CI −18.97 to −12.74, *p* < 0.05], and VAS MD −3.12 [95%CI −3.49 to −2.76, *p* < 0.05]. Subgroup analysis indicated a significant difference between follow-up periods in THI (*p* < 0.0001) and VAS loudness (*p* = 0.02). Conclusions: Cochlear implantation substantially improves tinnitus in patients with hearing loss, though the effect may diminish over time. Further research is needed to confirm these findings.

## 1. Introduction

Tinnitus is described as the perception of sound without any external source [1]. According to the American Tinnitus Association, around 20% of American adults have experienced persistent tinnitus for over 6 months [2], and over 6% have had severe tinnitus that significantly affects their quality of life [3]. Multiple factors contribute to tinnitus [4], among which the main risk factor is hearing loss (HL) [5]. It was reported that 67% of patients with single-sided sensorineural hearing loss (SNHL) experienced annoying tinnitus as measured by THI [6]. The most applied treatment for sensorineural hearing loss involves medical devices, including cochlear implants and hearing aids [7]. Cochlear implantation (CI) is widely used to address auditory impairment, especially in patients with poor speech recognition and advanced SNHL [8,9], including bilateral and single-sided cases with or without tinnitus [10]. Deklerck et al. reported promising results for cochlear implantation in tinnitus, with a total suppression rate of 60%. Notably, 80% of patients experienced a lasting reduction in tinnitus, even after the implant was removed [11]. However, in contradiction, Assouly et al. demonstrated that 9.2% of patients reported a new onset of tinnitus, and 2% had an increase of existing tinnitus after CI [12]. Previous studies have shown substantial improvement of CI on tinnitus in single-side-deafness (SSD) patients [13,14], noting no significant difference between long- and short-term follow-up, whose clinical implication needs further investigation.

Thus, to further ascertain the longitudinal impact of CI on tinnitus and explore the heterogeneity resource, we carried out this systematic review and meta-analysis. We included the studies on SNHL patients with tinnitus from 2005 to 2023, and the main results were THI (tinnitus index), TQ (tinnitus questionnaire), and VAS (visual analysis).

## 2. Materials and Methods

### 2.1. Search Strategy

A systematic review was conducted according to the Preferred Reporting Items for Systematic Reviews and Meta-Analyses (PRISMA) guidelines 2020 [15] and was registered in the Prospective Register of Systematic Reviews (PROSPERO) (CRD42024547608). A search was conducted in PubMed, Embase, and the Cochrane Library from inception to 30 April 2024. The search strategy included a combination of Medical Subject Heading terms (MeSH) and keywords. No language restrictions were applied in our review. The keywords of retrieval strategy included: (((cochlear implant) OR (auditory prosthesis) OR (cochlear prosthesis)) AND ((tinnitus) OR (ringing) OR (buzzing) OR (booming))). A manual search was conducted using the lists of references of key articles (see Supplementary Materials).

### 2.2. Inclusion and Exclusion Criteria

To be included in the meta-analyses, original studies had to meet the following criteria: (1) participants are adult patients (over 18) of either sex; (2) participants with severe to profound SNHL accompanied with stable tinnitus for more than 1 year that failed to respond to other treatments for tinnitus; (3) comparative data between pre- and postoperative states; (4) the outcomes included Tinnitus Handicap Inventory (THI), Visual Analog Scale (VAS), or Tinnitus Questionnaire (TQ).

The exclusion criteria are as follows: (1) studies are review articles, supplemental reports, case reports, and studies on basic science or animal studies, and studies include less than 2 patients; (2) studies included patients with vascular disorders, neurology disorders, and other systemic diseases that could generate tinnitus; (3) studies lack data integrity, such as postoperative outcomes, and exact data of mean and standard deviation; (4) studies by the same authors in overlapping populations (to prevent the same population were examined more than once in our review, we reported the more recent one between two studies by the same author).

### 2.3. Data Extraction

The search results were evaluated by two reviewers independently. First, all duplicates of the collected studies were removed. Then, irrelevant studies and studies with incomplete or missing statistical data were excluded. The full texts of the other articles were then carefully reviewed to ensure they met the eligibility criteria. Data in relevant studies were collected in a structured form. The data extraction process included the following variables: author, year, study types, countries, the total number of participants, gender, age, the most recent follow-up, duration of tinnitus, and relevant outcome measurements. Clinical outcomes extracted were VAS, TQ, and THI (mean score and standard deviation), both preoperative and postoperative.

Three types of tinnitus questionnaires were involved in tinnitus evaluation. The Tinnitus Handicap Inventory (THI, 0–100) was grouped by scores as follows: slight (0–16), mild (18–36), moderate (38–56), severe (58–76), or catastrophic (78–100). The Visual Analog Scale (VAS, 0–10) was designed as several 10-scale bands for participants to mark the degree they perceived tinnitus annoyance, loudness, effect on life, and awareness of tinnitus. The Tinnitus Questionnaire (TQ, 0–84), consisting of 52 questions, indicates the severity levels of distress [16,17]. Values were accurate to two decimal places.

#### 2.3.1. Meta-Analysis 

Meta-analysis was performed to assess the improvement before and after implantation using Review Manager (RevMan, version 5.3; The Cochrane Collaboration, London, UK). All scaled outcomes measuring tinnitus, including at least 2 independent studies, were quantitatively meta-analyzed. Additionally, we launched subgroup analyses based on single-sided/bilateral deafness, follow-up period, and the ethnicity of participants. We predicted high heterogeneity for the study characteristics (I^2^ < 25%, no heterogeneity; 25% ≤ I^2^ < 50%, low heterogeneity; 50% ≤ I^2^ < 75%, moderate heterogeneity; I^2^ > 75%, high heterogeneity) [18]. When a study with less than 50% heterogeneity, a fixed-effects model was performed; otherwise, a random-effects model was used, with mean differences with 95% confidence intervals (CI) and a *p*-value of 0.05 [19,20,21,22]. The forest plot was generated by Review Manager 5.42 (The Nordic Cochrane Centre, The Cochrane Collaboration, Copenhagen, Denmark). 

#### 2.3.2. Evaluation of the Bias

Two reviewers independently assess the risk of bias based on Joanna Briggs’s Critical Appraisal Checklist for Case Series and Cohort Studies [23]. Guidelines for evaluation criteria were as follows: clear criteria for inclusion; valid methods for identification of condition for participants; complete inclusion of participants; standardized measurement of condition; consecutive inclusion of participants; and clear reporting of demographics, participants’ clinical information, outcomes, and follow-ups, presenting sites, and statistical analysis.

## 3. Results

### 3.1. Search Results

**Figure 1:**
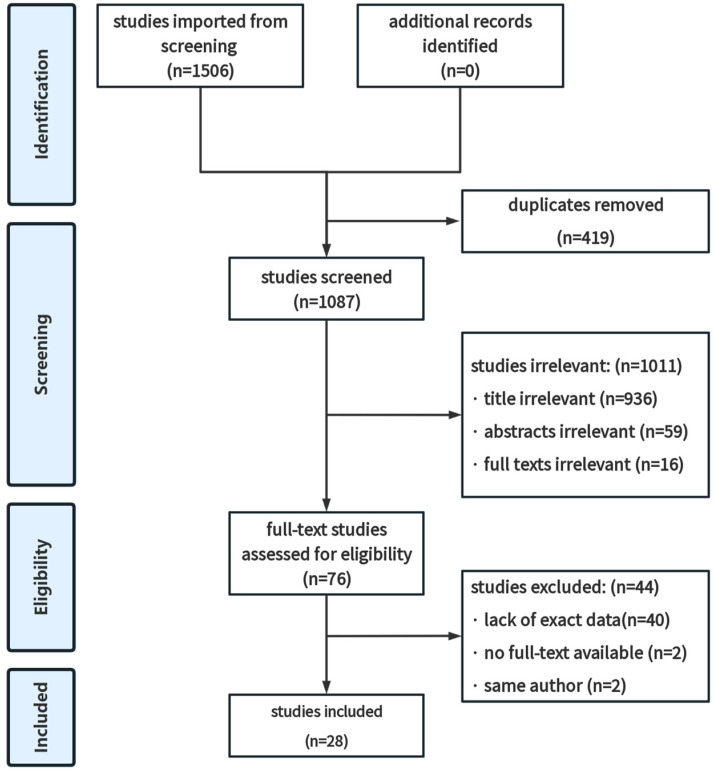
PRISMA flow diagram of identification, screening, eligibility, and inclusion of studies.

**Figure 2:**
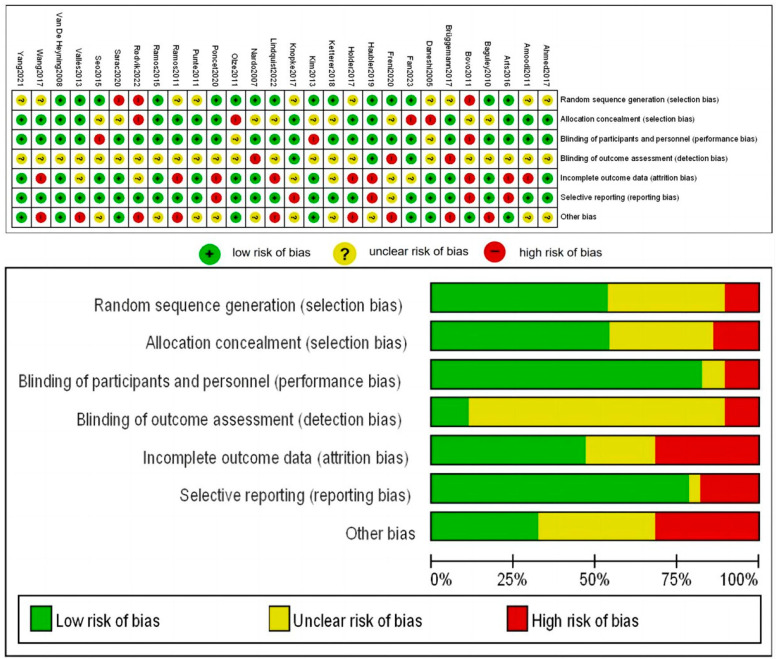
Risk of bias for 28 studies [24,25,26,27,28,29,30,31,32,33,34,35,36,37,38,39,40,41,42,43,44,45,46,47,48,49,50,51].

Two reviewers independently identified 1506 studies, and 419 duplicates were removed (Figure 1). Then, 1087 articles were screened by irrelevant titles, abstracts excluded 59, and full texts excluded 16. Among the remaining 76 articles, 40 lack exact data, and 2 have no full text available. Ultimately, 2 articles by the same authors were excluded, and the most recent one was selected. (Two articles by Punte were included as they possess different tinnitus outcomes). Among 28 studies induced were published between 2010 and 2023, 20 were prospective cohorts [24,25,26,27,28,29,30,31,32,33,34,35,36,37,38,39,40,41,42,43], 1 were case series [44], and 7 were retrospective studies [45,46,47,48,49,50,51] (Table 1). Moreover, 11 of the studies focused on single-sided-deafness [25,26,29,30,35,37,38,42,44,45,48], and 19 studies were primarily conducted in Europe [26,27,28,30,31,32,34,35,36,37,38,39,40,42,45,46,47,49,50]. The risk of bias was evaluated for each included study (Figure 2).

**Table 1:** Characteristics of the trials included in the systematic review and meta-analyses.

First Author	Year	Study Type	Country	No. ^a^	Mean Age, Y	Sex, M:F	Most Recent Follow-Up, Mon	Duration of Tinnitus, Y	Relevant Outcome Measures
Ahmed [45]	2017	Retrospective	Egypt	13	40 ± 10	8/5	3	**^b^**	THI, TRS
Amoodi [24]	2011	Prospective	Canada	142	54.2 ± 14.68	57/87	12		THI, HHI, F36
Arts [25]	2016	Prospective	The Netherlands	9/10			3	3	THI, TQ, VAS
Baguley [26]	2010	Prospective	UK	21			24		TQ, VAS
Bovo [27]	2011	Prospective	Italy	36/51	46 ± 17.5	17/34	6		THI
Brüggemann [28]	2017	Prospective	Germany	47	58.62	17/30	12		TQ
Daneshi [46]	2015	Retrospective	Iran	20/52	28.85	12/8	1.7	90.19 ± 92.81	TQ
Fan [29]	2023	Prospective	China	7			6		THI, VAS
Freni [47]	2020	Retrospective	France	98	54.35 ± 11.34	52/46	12		THI, VAS
Haubler [30]	2019	Prospective	Germany	21			18	4.8 ± 7.7	TQ
Holder [48]	2017	Retrospective	USA	12	51.6	10/2	6		THI
Ketterer [31]	2018	Prospective	Germany	44	62.7 ± 12.84	24/20	12		TQ
Knopke [32]	2017	Prospective	Germany	41	61 ± 13.45		24		TQ, NCIQ, OI
Kim [33]	2013	Prospective	Asia	22/35	47.5 ± 15.1	11/11	10.5	13.6 ± 13.7	THI
Nardo [49]	2007	Retrospective	Italy	20	43.33 ± 15.75		6		THI
Olze [34]	2011	Prospective	Germany	43/58	51.7 ± 16.9	12/31	9		HRQoL, TQ
Lindquist [44]	2022	Case series	USA	23			6		THI
Poncet [35]	2020	Prospective	France	23/26	54.2 ± 10	14/9	13	7.2 ± 5	THI, TQ, VAS,
Punte [36]	2011	Prospective	Belgium	26			12		VAS
Ramos [38]	2015	Prospective	Switzerland	13/16	53.1	6/7	12		VAS
Ramos [37]	2011	Prospective	Spain	4/10			18		THI, VAS
Rødvik [39]	2022	Prospective	Norway	12/20	47.4 ± 15.0		24		THI, VAS, SRQ
Sarac [40]	2020	Prospective	Turkey	23	42.5 ± 15.9	13/10	6		THI, BDI
Seo [41]	2015	Prospective	South Korea	16	51.94 ± 13.73	10/6	6		THI
Vallés [50]	2013	Retrospective	Spain	20			12		VAS
Van De Heyning [42]	2008	Prospective	Belgium	12/22			24		TQ
Wang [43]	2017	Prospective	China	21	53.52 ± 14.25	8/13	12		THI, VAS
Yang [51]	2021	Retrospective	China	51	41.0 ± 17.0	24/27	18		THI, VAS

Abbreviations: THI, Tinnitus Handicap Inventory; TRS, Tinnitus Rating Scale; TQ, Tinnitus Questionnaire; VAS, visual analog score, NCIQ, Nijmegen Cochlear Implantation Questionnaire; OI, Oldenburg Inventory; SRQ, a self-report questionnaire. ^a^: participants in outcomes/patients meeting inclusion criteria. ^b^: a blank cell means there are no specific data reported in the study.

### 3.2. Cohort Characteristics

A total of 26 studies consisted of 741 patients with a mean age of 36.2 to 62.7, a mean tinnitus duration of 7.2 to 9.63 years, and a follow-up time from 3 to 24 months. In studies specifying sex, 280 were male (53.4%), and 244 (46.6%) were female (Table 1). For tinnitus measurements, 16 studies applied THI, 9 TQ, and 10 VAS. Among the studies specified, 186 patients possessed single-sided deafness.

### 3.3. Questionnaire Outcomes

#### 3.3.1. THI Outcomes

**Figure 3:**
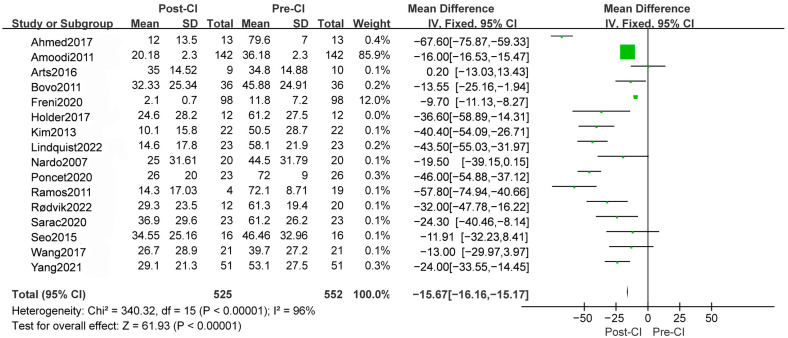
A total outcome of forest plots from a meta-analysis of THI [24,25,33,35,37,39,40,41,43,44,45,47,48,49,51].

Eleven studies (50%) recorded THI outcomes, including 282 patients, demonstrating a significant reduction in THI scores. CI resulted in a mean difference (MD) of −13.03 [95%CI, −14.37 to −11.69, *p* < 0.00001] (Figure 3). According to 5 studies specifying single-sided deafness among them, THI resulted in an MD of −46.34 [95%CI, −51.19 to −41.49, *p* < 0.00001], with a significant difference compared to total deaf patients (*p* = 0.03), whose MD is −11.95 [95%CI, −22.61 to −1.28, *p* = 0.006] (Figure S1A). Moreover, THI results remain significant when stratified by follow-up time, with an MD of −48.56 [95%CI, −55.57 to −41.55, *p* < 0.00001], −35.94 [95%CI, −45.08 to −26.79, *p* < 0.00001] (Figure S1C), and −10.64 [95%CI, −12.05 to −9.23, *p* < 0.00001], respectively, in 3 months, 6 months and more than 1 year, compared to baseline, with significant differences between subgroups (*p* < 0.00001). For there is no overlapping CI between subgroups, the difference in follow-up time is considered significant, which suggests that the effects of CI in THI may change over time. In the subgroup analysis of different continents, the MD of THI is −36.20 [95%CI, −64.94 to −7.45, *p* < 0.00001] in European studies, −20.00 [95%CI, −27.70 to −12.30, *p* < 0.00001] in Asian studies, and −42.04 [95%CI, −52.29 to −31.80, *p* < 0.00001], with a significant difference between subgroups (Figure S1D). Meanwhile, heterogeneity is dramatically reduced in subgroup analysis of Asian and North American studies. In conclusion, CI remarkably improves tinnitus in THI scores, whose effect differs in different follow-up periods, and part of the heterogeneity comes from different regions.

#### 3.3.2. TQ Outcomes

**Figure 4:**
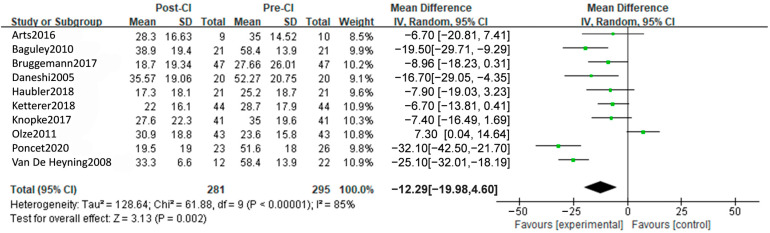
A total outcome of forest plot from the meta-analysis of TQ [25,26,28,30,31,32,34,35,42,46].

Meta-analysis of 8 studies in 218 patients demonstrated a significant improvement in TQ with an MD of −14.87 [95%CI, −18.09 to −11.65, *p* < 0.00001] (Figure 4). Six studies focused on SSD indicated a result with an MD of −22.07 [95%CI: −25.28 to −16.59, *p* < 0.0001], while the MD of 2 studies in BHL is −8.16 [95%CI: −14.65, −1.68], with a significant difference between subgroups (*p* = 0.001) (Figure S2A). When analyzed by follow-up period, reduction in TQ scores maintained significant at short-term follow-up (3, 12, 13 months), MD = −15.63 [95%CI, −20.46 to −10.81, *p* < 0.0001] and long-term follow-up (18, 24 months), MD = −12.22 [95%CI, −16.94 to −7.51, *p* < 0.0001] relative to baseline, with no significant difference between subgroups (Figure S2B). In summary, CI has a notable decrease in TQ among tinnitus patients.

#### 3.3.3. VAS Outcomes

**Figure 5:**
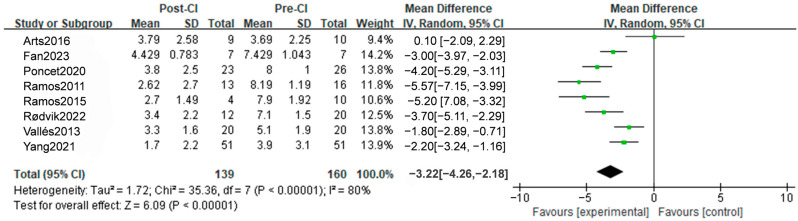
A total outcome of forest plots from the meta-analysis of VAS [25,29,35,37,38,39,50,51].

In eight studies involving 139 participants, it was found that CI resulted in a mean VAS difference of −3.12 [95%CI, −3.57 to −22.68, *p* < 0.0001] (Figure 5). The analysis showed a significant decrease in VAS in different follow-up times, with an MD of −2.51 [95%CI, −3.40 to −1.63, *p* < 0.0001], −3.47 [95%CI, −4.17 to−2.78, *p* < 0.0001], and −2.97 [95%CI, −3.78, −2.17, *p* < 0.0001], respectively, in short-term (3, 6 months), middle-term (12, 13 months) and long-term follow-up (18, 24 months), with no significant difference between subgroups (*p* = 0.24) (Figure S3A). The results remained significant in region subgroups, with an MD of −3.63 [95%CI, −4.24 to −3.01, *p* < 0.05] in Europe and −2.63 [95%CI, −3.34 to −1.92, *p* < 0.05] in Caucasians, revealing a subgroup difference (*p* = 0.0004) (Figure S3B). Furthermore, a meta-analysis of four studies including only SSD patients showed a significant effect, with an MD of −3.42 [95%CI, −4.05 to −2.79, *p* < 0.05] (Figure S3C). In summary, CI has a notable impact on decreasing VAS scores.

### 3.4. Assessment of Studies with Multiple Follow-Up Periods

**Figure 6:**
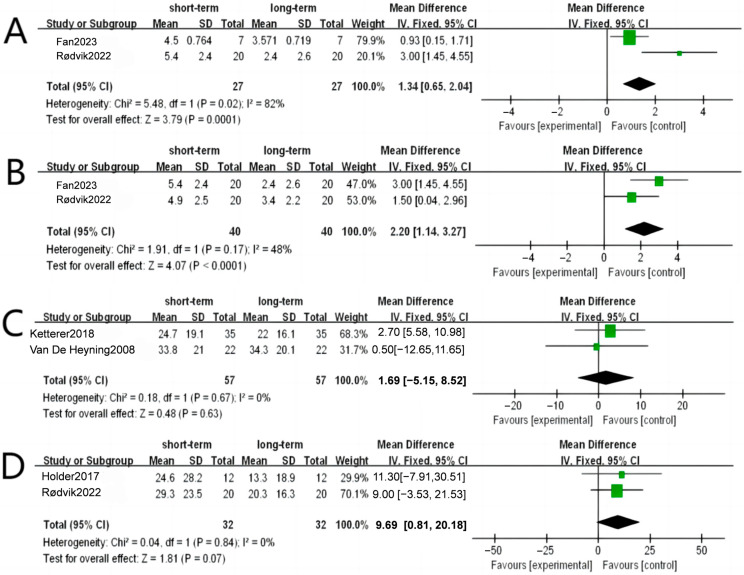
Forest plots from the meta-analysis studies with multiple follow-up periods: (**A**) outcomes of tinnitus loudness, (**B**) outcomes of tinnitus annoyance, (**C**) outcomes of TQ, and (**D**) outcomes of THI. CI, confidence interval [29,31,39,42,48].

According to 5 studies analyzing different follow-up periods, a significant difference was found in tinnitus loudness in VAS between short-term follow-up and long-term follow-up, with an MD of −3.47 1.34 [95%CI: 0.65 to 2.04, *p* = 0.02]. In contrast, other indications, including THI, TQ, and tinnitus annoyance, showed no difference between short- and long-term follow-up (Figure 6).

### 3.5. Sensitivity Analysis

We conducted sensitivity analyses to evaluate the stability of our results. The detailed findings of these analyses, including the exclusion of high-risk bias studies and variations in statistical models, are provided in the Supplementary Materials (Table S2). In summary, while most analyses confirmed the robustness of our findings, some variability was noted when applying different effect models, which suggests a moderate influence of study heterogeneity on the overall outcome.

## 4. Discussion

The systematic review and meta-analysis demonstrate the positive effectiveness of cochlear implantation on tinnitus among patients with hearing loss. According to previous studies, CI is associated with significant improvements in auditory outcomes in SSD patients [52,53]. Meanwhile, the long-term effect (12 months) of CI was confirmed in both disabling [13] and SSD patients [54] with tinnitus. Consistent with previous studies [10,40], we found significant improvements in patients with tinnitus after CI as measured by three questionnaires assessing tinnitus annoyance, loudness, and impact on quality of life. Compared to previous efforts, we significantly increased the number of included studies and additionally performed subgroup analyses based on population race, follow-up period, and single-sided hearing loss, therefore broadening the scope of our outcomes. However, in contrast with previous studies, we found significant differences in subgroup analyses of follow-up periods in the Tinnitus Handicap Inventory (THI) (*p* < 0.00001), with no overlapping CI between subgroups, suggesting that the effect of CI on tinnitus might decrease over time, while the Tinnitus Questionnaire (TQ) and Visual Analog Scale (VAS) showed no significant differences between subgroups. We also analyzed studies with multiple follow-up periods, which showed a significant increase in tinnitus loudness as measured by VAS. Thus, more evidence is expected to further determine the longitudinal benefits of this intervention. 

There are no objective assessments for most cases of tinnitus [1]; thus, several questionnaires, including THI, VAS, and TQ, which investigate the subjective feelings of patients, are applied in our review. The Tinnitus Handicap Inventory (THI) assesses the physical, emotional, and functional effects of tinnitus on a patient [16]. The Visual Analog Scale (VAS) is a tool consisting of a line that separates several degrees of a phenomenon to visualize subjective perceptions [55]. When applied to tinnitus, it measures tinnitus loudness, annoyance, awareness, mental stress, and disability [56]. The Tinnitus Questionnaire (TQ) provides disease-specific outcomes and quality-of-life measurements for tinnitus patients [57]. Although these questionnaires indicate comprehensive changes in tinnitus, a statistically significant change may not identify the actual benefit perceived by patients [58]. Therefore, the Tinnitus Functional Index (TFI), with its sensitive responsiveness to changes in treatment, making it useful in clinical settings, was established [59]. Meanwhile, a standard is also expected to be set for evaluating tinnitus treatment [58].

This review indicates a significant positive outcome on tinnitus perception in hearing loss patients after CI, suggesting its usage in tinnitus patients with both bilateral and single-sided hearing loss. However, it must be acknowledged that a fraction of patients might experience worsened tinnitus or even new onset tinnitus. Given our results demonstrating differences in CI treatment effectiveness over time, regular follow-up is expected. 

Although comprehensive, there are several limitations in this review. First, the inclusion criteria, surgical methods, and outcome assessments are inconsistent, leading to high heterogeneity that prevents us from performing a reliable meta-analysis. Moreover, there is no available complete data on specific patient details, such as the hearing status of bilateral ears, duration of tinnitus, and tinnitus etiology, for us to further evaluate the effect of CI on individuals. Furthermore, the studies we included were mostly prospective cohort and clinical series, which necessitate more randomized controlled trials. Moreover, due to a lack of sufficient eligible studies, the China National Knowledge Infrastructure (CNKI) database was excluded from our search, potentially limiting the representation of research conducted in China or published in Chinese. Additionally, we did not perform subgroup analyses by language due to the limited number of non-English studies, which may obscure language-related biases. Lastly, our meta-analysis on follow-up time was limited by including only two studies, which affects the robustness and generalizability of our findings.

Thus, we were unable to analyze particular types of CI. For instance, there are numerous variables in CI, including implantation path, device selection, and programming, whose effects remain unseen due to the lack of information. Lastly, although significant improvements were found in THI, TQ, and VAS, we cannot prove their clinical significance; hence, the use of standard assessments of tinnitus, such as the Tinnitus Functional Index (TFI), needs to be promoted [58].

## 5. Conclusions

CI is an effective treatment for tinnitus, as assessed by THI, TQ, and VAS. Both single-sided and bilateral hearing loss CI users experienced significant improvement in tinnitus perception. We propose that tinnitus should be considered an important indication for CI. However, its benefits may diminish over time, and further research is expected to confirm these findings.

## Data Availability

The data supporting the findings of this study are available from publicly accessible databases and published literature. All data sources are cited within the article, and the datasets can be accessed through the corresponding references.

## Supplementary Materials

The following supporting information can be downloaded at: https://www.mdpi.com/article/10.3390/diagnostics14182028/s1, Table S1: Search Strategy; Figure S1: Outcomes of forest plots from the meta-analysis of THI; Figure S2: Outcomes of forest plots from the meta-analysis of TQ; Figure S3: Outcomes of forest plots from the meta-analysis of THI.
