# Supplementary material for: The Long-Term Effect of Cochlear Implantation on Tinnitus: A Systematic Review and Meta-Analysis
Source: Diagnostics (Basel). 2024 Sep 13;14(18):2028. doi: 10.3390/diagnostics14182028 (PMC11431415; doi:10.3390/diagnostics14182028)
Supplement: Supplementary file 1 [file diagnostics-14-02028-s001.zip › diagnostics-3165905-supplementary.pdf]

Table S1 Search Strategy

Cochran Search Strategy	53
#1	MeSH descriptor: [Cochlear Implants] explode all trees
#2	MeSH descriptor: [Tinnitus] explode all trees
#3	(tinnitus):ti,ab,kw
#4	(ringing):ti,ab,kw
#5	(buzzing):ti,ab,kw
#6	#3 OR #4 OR #5
#7	(cochlear implant):ti,ab,kw
#8	(cochlear implantation):ti,ab,kw
#9	(implant, cochlear):ti,ab,kw
#10	(cochlear prosthesis):ti,ab,kw
#11	(cochlear prostheses):ti,ab,kw
#12	(prostheses, cochlear):ti,ab,kw
#13	(prosthesis, cochlear):ti,ab,kw
#14	(auditory prosthesis):ti,ab,kw
#15	(auditory prostheses):ti,ab,kw
#16	(auditory, prostheses):ti,ab,kw
#17	(auditory, prosthesis):ti,ab,kw
#18	(prostheses, auditory):ti,ab,kw
#19	(prosthesis, auditory):ti,ab,kw
#20	#1 OR #7 OR #8 OR #9 OR #10 OR #11 OR #12 OR #13 OR #14 OR #15 OR #16 OR #17 OR #18 OR #19
#21	#6 AND #20
PubMed Search Strategy	561
(((((tinnitus[Title/Abstract]) OR (Ringing[Title/Abstract])) OR (Buzzing[Title/Abstract])) OR (Booming[Title/Abstract])) OR ("Tinnitus"[Mesh])) AND (("Cochlear Implants"[Mesh]) OR ((((((((((((cochlear implant[Title/Abstract]) OR (cochlear implantation[Title/Abstract])) OR (implant, cochlea[Title/Abstract])) OR (cochlear prosthesis[Title/Abstract])) OR (cochlear prosthesis[Title/Abstract])) OR (cochlear prostheses[Title/Abstract])) OR (cochlear prostheses[Title/Abstract])) OR (prostheses, cochlear[Title/Abstract])) OR (prosthesis, cochlear[Title/Abstract])) OR (auditory prosthesis[Title/Abstract])) OR (auditory prostheses[Title/Abstract])) OR (prostheses, auditory[Title/Abstract])) OR (prosthesis, auditory[Title/Abstract])))	
Embase Search Strategy	892
#1	cochlear implant'/exp OR 'cochlear implant':ab,ti OR 'implant, cochlear':ab,ti OR 'cochlear prosthesis':ab,ti OR 'cochlear prostheses':ab,ti OR 'cochlear prostheses':ab,ti OR 'prostheses, cochlear':ab,ti OR 'prosthesis, cochlear':ab,ti OR 'auditory prosthesis':ab,ti OR 'auditory prosthesis':ab,ti OR 'auditory prostheses':ab,ti OR 'prostheses, auditory':ab,ti OR 'prosthesis, auditory':ab,ti
#2	tinnitus'/exp OR tinnitus:ab,ti OR ringing:ab,ti OR buzzing:ab,ti OR booming
#3	#1 AND #2

Table S2 Outcomes of Sensitivity analysis.SSD, single-side-deafness.

	THI-total		TQ-total		VAS-total	
**Analysis Scenario**	**Pooled Effect Size (95% CI)**	**Heterogeneity (I²)**	**Pooled Effect Size (95% CI)**	**Heterogeneity (I²)**	**Pooled Effect Size (95% CI)**	**Heterogeneity (I²)**
Including all studies	-15.67 [-16.16, -15.17]	96%	-11.59 [-14.45, -8.72]	85%	-3.12 [-3.57, -2.68]	80%
Excluding high-risk of bias studies	-16.34 [-16.87, -15.81]	96%	-10.22 [-13.40, -7.05]	87%	-2.64 [-3.20, -2.07]	72%
Random-effects model	-28.43 [-33.96, -22.90]	96%	-12.29 [-19.98, -4.60]	85%	-3.22 [-4.26, -2.18]	80%
Fixed-effects model	-15.67 [-16.16, -15.17]	96%	-11.59 [-14.45, -8.72]	85%	-3.12 [-3.57, -2.68]	80%

	THI-SSD		TQ-SSD		VAS-region	
**Analysis Scenario**	**Pooled Effect Size (95% CI)**	**Heterogeneity (I²)**	**Pooled Effect Size (95% CI)**	**Heterogeneity (I²)**	**Pooled Effect Size (95% CI)**	**Heterogeneity (I²)**
Including all studies	-13.01 [-14.37, -11.66]	98%	-16.98 [-20.59, -13.37]	76%	-3.20 [-3.66, -2.74]	76%
Excluding high-risk of bias studies	-36.06 [-65.51, -6.60]	99%	-16.71 [-21.00, -12.41]	76%	-2.99 [-3.65, -2.34]	86%
Random-effects model	-32.38 [-50.91, -13.85]	98%	-15.75 [-23.38, -8.13]	76%	-3.50 [-4.48, -2.52]	76%
Fixed-effects model	-13.01 [-14.37, -11.66]	98%	-16.98 [-20.59, -13.37]	76%	-3.20 [-3.66, -2.74]	76%

	THI-region		TQ-follow-up time		THI-follow-up time	
**Analysis Scenario**	**Pooled Effect Size (95% CI)**	**Heterogeneity (I²)**	**Pooled Effect Size (95% CI)**	**Heterogeneity (I²)**	**Pooled Effect Size (95% CI)**	**Heterogeneity (I²)**
Including all studies	-15.56 [-16.05, -15.06]	96%	-10.57 [-13.55, -7.60]	84%	-3.07 [-3.52, -2.61]	79%
Excluding high-risk of bias studies	-33.31 [-50.25, -16.37]	96%	-12.47 [-21.85, -3.09]	82%	-2.89 [-3.44, -2.35]	81%
Random-effects model	-27.79 [-33.61, -21.96]	96%	-10.26 [-17.98, -2.54]	82%	-3.18 [-4.21, -2.15]	79%
Fixed-effects model	-15.56 [-16.05, -15.06]	96%	-10.57 [-13.55, -7.60]	84%	-3.07 [-3.52, -2.61]	79%

	THI-follow-up time	
**Analysis Scenario**	**Pooled Effect Size (95% CI)**	**Heterogeneity (I²)**
Including all studies	-15.67 [-16.16, -15.17]	96%
Excluding high-risk of bias studies	-16.06 [-16.60, -15.53]	87%
Random-effects model	-28.40 [-33.93, -22.87]	96%
Fixed-effects model	-15.67 [-16.16, -15.17]	96%

Figure S1 Outcomes of forest plots from the meta−analysis of THI: (A) subgroup analysis for SSD and BHL, (B) subgroup analysis for follow-up period, and (C) subgroup analysis for continents. CI, confidence interval; SSD, single-side-deafness; BHL, bilateral hearing loss.

	Post-CI			Pre-CI				Mean Difference
Study or Subgroup	Mean	SD	Total	Mean	SD	Total	Weight	IV, Fixed, 95% CI
**1.3.1 SSD**								
Ahmed2017	12	13.5	13	79.6	7	13	2.7%	-67.60 [-75.87, -59.33]
Arts2016	35	14.52	9	34.8	14.88	10	1.0%	0.20 [-13.03, 13.43]
Holder2017	24.6	28.2	12	61.2	27.5	12	0.4%	-36.60 [-58.89, -14.31]
Lindquist2022	14.6	17.8	23	58.1	21.9	23	1.4%	-43.50 [-55.03, -31.97]
Poncet2020	26	20	23	72	9	26	2.3%	-46.00 [-54.88, -37.12]
**Subtotal (95% CI)**			**80**			**84**	**7.8%**	**-46.34 [-51.19, -41.49]**
Heterogeneity: Chi² = 73.92, df = 4 (P < 0.00001); I² = 95%								
Test for overall effect: Z = 18.74 (P < 0.00001)								
**1.3.2 BHL**								
Freni2020	2.1	0.7	98	11.8	7.2	98	89.4%	-9.70 [-11.13, -8.27]
Rødvik2022	29.3	23.5	12	61.3	19.4	20	0.7%	-32.00 [-47.78, -16.22]
Yang2021	29.1	21.3	51	53.1	27.5	51	2.0%	-24.00 [-33.55, -14.45]
**Subtotal (95% CI)**			**161**			**169**	**92.2%**	**-10.19 [-11.60, -8.78]**
Heterogeneity: Chi² = 15.82, df = 2 (P = 0.0004); I² = 87%								
Test for overall effect: Z = 14.16 (P < 0.00001)								
**Total (95% CI)**			**241**			**253**	**100.0%**	**-13.01 [-14.37, -11.66]**
Heterogeneity: Chi² = 286.75, df = 7 (P < 0.00001); I² = 98%								
Test for overall effect: Z = 18.83 (P < 0.00001)								
Test for subgroup differences: Chi² = 197.01, df = 1 (P < 0.00001), I² = 99.5%								

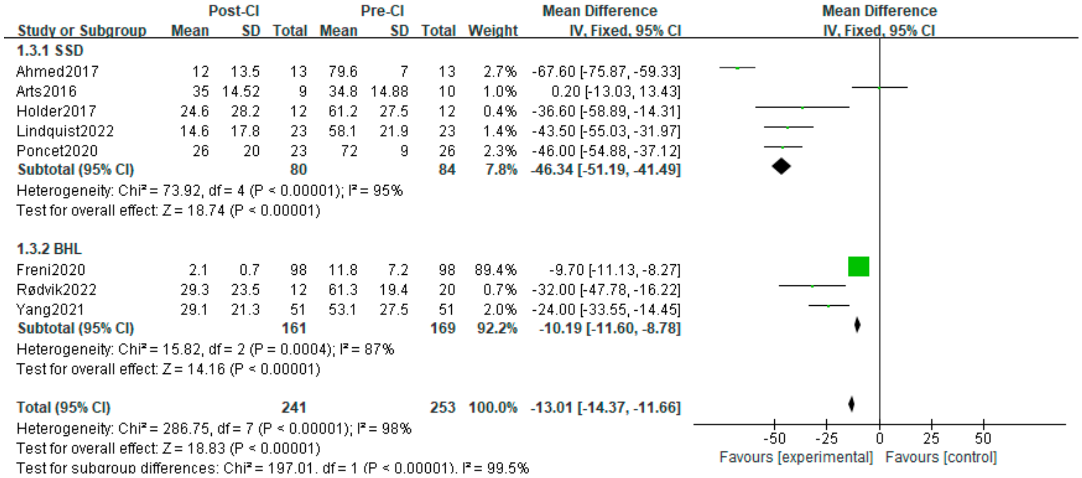

(A)

	Post-CI			Pre-CI				Mean Difference
Study or Subgroup	Mean	SD	Total	Mean	SD	Total	Weight	IV, Fixed, 95% CI
**1.4.1 3M**								
Ahmed2017	12	13.5	13	79.6	7	13	0.4%	-67.60 [-75.87, -59.33]
Arts2016	35	14.52	9	34.8	14.88	10	0.1%	0.20 [-13.03, 13.43]
**Subtotal (95% CI)**			**22**			**23**	**0.5%**	**-48.56 [-55.57, -41.55]**
Heterogeneity: Chi² = 72.56, df = 1 (P < 0.00001); I² = 99%								
Test for overall effect: Z = 13.58 (P < 0.00001)								
**1.4.3 6M**								
Bovo2011	32.33	25.34	36	45.88	24.91	36	0.2%	-13.55 [-25.16, -1.94]
Holder2017	24.6	28.2	12	61.2	27.5	12	0.0%	-36.60 [-58.89, -14.31]
Kim2013	10.1	15.8	22	50.5	28.7	22	0.1%	-40.40 [-54.09, -26.71]
Lindquist2022	14.6	17.8	23	58.1	21.9	23	0.2%	-43.50 [-55.03, -31.97]
Nardo2007	25	31.61	20	44.5	31.79	20	0.1%	-19.50 [-39.15, 0.15]
Sarac2020	36.9	29.6	23	61.2	26.2	23	0.1%	-24.30 [-40.46, -8.14]
Seo2015	34.55	25.16	16	46.46	32.96	16	0.1%	-11.91 [-32.23, 8.41]
**Subtotal (95% CI)**			**152**			**152**	**0.8%**	**-28.56 [-34.23, -22.90]**
Heterogeneity: Chi² = 19.91, df = 6 (P = 0.003); I² = 70%								
Test for overall effect: Z = 9.88 (P < 0.00001)								
**1.4.4 more than1Y**								
Amoodi2011	20.18	2.3	142	36.18	2.3	142	85.9%	-16.00 [-16.53, -15.47]
Freni2020	2.1	0.7	98	11.8	7.2	98	12.0%	-9.70 [-11.13, -8.27]
Poncet2020	26	20	23	72	9	26	0.3%	-46.00 [-54.88, -37.12]
Ramos2015	14.3	17.03	4	72.1	8.71	10	0.1%	-57.80 [-75.34, -40.26]
Rødvik2022	29.3	23.5	12	61.3	19.4	20	0.1%	-32.00 [-47.78, -16.22]
Wang2017	26.7	28.9	21	39.7	27.2	21	0.1%	-13.00 [-29.97, 3.97]
Yang2021	29.1	21.3	51	53.1	27.5	51	0.3%	-24.00 [-33.55, -14.45]
**Subtotal (95% CI)**			**351**			**368**	**98.7%**	**-15.40 [-15.90, -14.90]**
Heterogeneity: Chi² = 141.23, df = 6 (P < 0.00001); I² = 96%								
Test for overall effect: Z = 60.48 (P < 0.00001)								
**Total (95% CI)**			**525**			**543**	**100.0%**	**-15.67 [-16.16, -15.17]**
Heterogeneity: Chi² = 339.28, df = 15 (P < 0.00001); I² = 96%								
Test for overall effect: Z = 61.92 (P < 0.00001)								
Test for subgroup differences: Chi² = 105.59, df = 2 (P < 0.00001), I² = 98.1%								

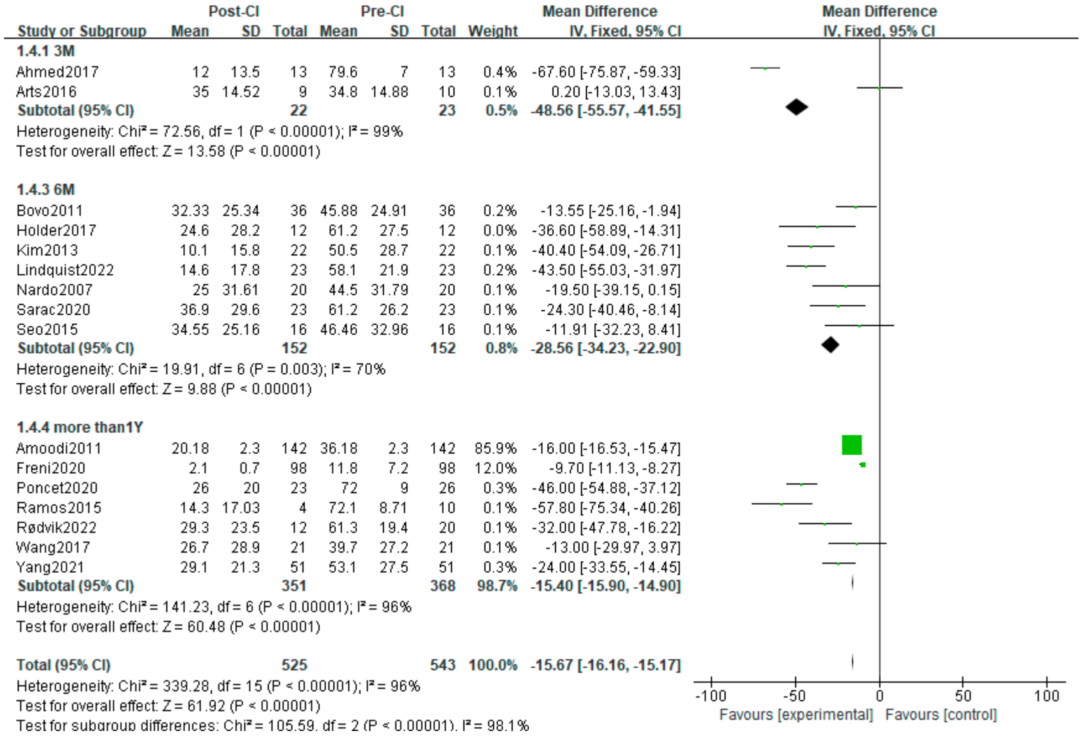

(B)

Study or Subgroup	Post-CI Mean	Post-CI SD	Post-CI Total	Pre-CI Mean	Pre-CI SD	Pre-CI Total	Weight	Mean Difference IV, Random, 95% CI
**1.5.1 Europe**								
Ahmed2017	12	13.5	13	79.6	7	13	9.0%	-67.60 [-75.87, -59.33]
Bovo2011	32.33	25.34	36	45.88	24.91	36	7.6%	-13.55 [-25.16, -1.94]
Freni2020	2.1	0.7	98	11.8	7.2	98	10.9%	-9.70 [-11.13, -8.27]
Nardo2007	25	31.61	20	44.5	31.79	20	4.9%	-19.50 [-39.15, 0.15]
Poncet2020	26	20	23	72	9	23	8.7%	-46.00 [-54.96, -37.04]
Ramos2011	14.3	17.03	4	72.1	8.71	4	5.1%	-57.80 [-76.55, -39.05]
Rødvik2022	29.3	23.5	12	61.3	19.4	20	6.1%	-32.00 [-47.78, -16.22]
Sarac2020	36.9	29.6	23	61.2	26.2	23	5.9%	-24.30 [-40.46, -8.14]
**Subtotal (95% CI)**			**229**			**237**	**58.3%**	**-33.78 [-53.44, -14.12]**
Heterogeneity: Tau² = 756.37; Chi² = 269.75, df = 7 (P < 0.00001); I² = 97%								
Test for overall effect: Z = 3.37 (P = 0.0008)								
**1.5.2 Asia**								
Seo2015	34.55	25.16	16	46.46	32.96	16	4.7%	-11.91 [-32.23, 8.41]
Wang2017	26.7	28.9	21	39.7	27.2	21	5.7%	-13.00 [-29.97, 3.97]
Yang2021	29.1	21.3	51	53.1	27.5	51	8.5%	-24.00 [-33.55, -14.45]
**Subtotal (95% CI)**			**88**			**88**	**18.9%**	**-20.00 [-27.70, -12.30]**
Heterogeneity: Tau² = 0.00; Chi² = 1.94, df = 2 (P = 0.38); I² = 0%								
Test for overall effect: Z = 5.09 (P < 0.00001)								
**1.5.3 North America**								
Amoodi2011	20.18	2.3	142	36.18	2.3	142	11.0%	-16.00 [-16.53, -15.47]
Holder2017	24.6	28.2	12	61.2	27.5	12	4.2%	-36.60 [-58.89, -14.31]
Lindquist2022	14.6	17.8	23	58.1	21.9	23	7.7%	-43.50 [-55.03, -31.97]
**Subtotal (95% CI)**			**177**			**177**	**22.9%**	**-31.09 [-52.64, -9.54]**
Heterogeneity: Tau² = 315.55; Chi² = 25.05, df = 2 (P < 0.00001); I² = 92%								
Test for overall effect: Z = 2.83 (P = 0.005)								
**Total (95% CI)**			**494**			**502**	**100.0%**	**-29.45 [-35.28, -23.63]**
Heterogeneity: Tau² = 80.35; Chi² = 317.58, df = 13 (P < 0.00001); I² = 96%								
Test for overall effect: Z = 9.92 (P < 0.00001)								
Test for subgroup differences: Chi² = 2.28, df = 2 (P = 0.32), I² = 12.1%								

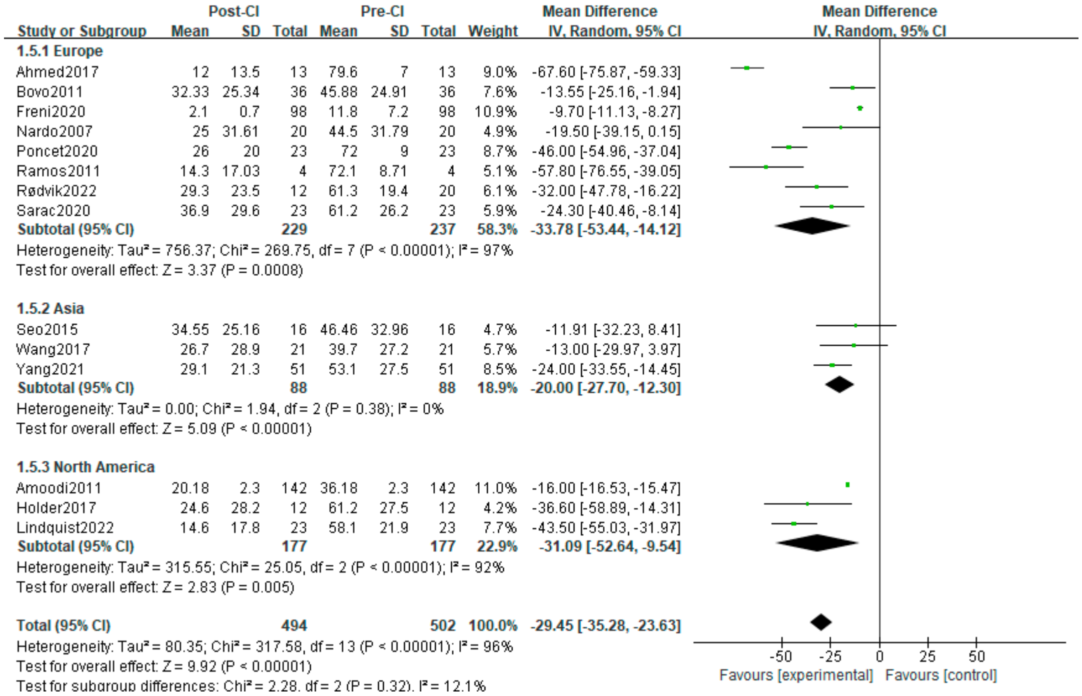

(C)

Figure S2 Outcomes of forest plots from the meta−analysis of TQ: (A) subgroup analysis for single-side deafness, and (B)subgroup analysis of the follow-up period. CI, confidence interval; SSD, single-side-deafness; BHL, bilateral hearing loss.

Study or Subgroup	Post-CI Mean	Post-CI SD	Post-CI Total	Pre-CI Mean	Pre-CI SD	Pre-CI Total	Weight	Mean Difference IV, Random, 95% CI
**2.7.1 SSD**								
Arts2016	28.3	16.63	9	35	14.52	10	11.6%	-6.70 [-20.81, 7.41]
Baguley2010	38.9	19.4	21	58.4	13.9	21	14.2%	-19.50 [-29.71, -9.29]
Haubler2019	17.3	18.1	21	25.2	18.7	21	13.6%	-7.90 [-19.03, 3.23]
Poncet2020	19.5	19	23	51.6	18	26	14.1%	-32.10 [-42.50, -21.70]
Van De Heyning2008	33.3	6.6	12	58.4	13.9	22	16.5%	-25.10 [-32.01, -18.19]
**Subtotal (95% CI)**			**86**			**100**	**70.0%**	**-19.04 [-27.88, -10.20]**
Heterogeneity: Tau² = 72.96; Chi² = 15.08, df = 4 (P = 0.005); I² = 73%								
Test for overall effect: Z = 4.22 (P < 0.0001)								
**2.7.2 BHL**								
Brüggemann2017	18.7	19.34	47	27.66	26.01	47	14.9%	-8.96 [-18.23, 0.31]
Knopke2017	27.6	22.3	41	35	19.6	41	15.0%	-7.40 [-16.49, 1.69]
**Subtotal (95% CI)**			**88**			**88**	**30.0%**	**-8.16 [-14.65, -1.68]**
Heterogeneity: Tau² = 0.00; Chi² = 0.06, df = 1 (P = 0.81); I² = 0%								
Test for overall effect: Z = 2.47 (P = 0.01)								
**Total (95% CI)**			**174**			**188**	**100.0%**	**-15.75 [-23.38, -8.13]**
Heterogeneity: Tau² = 79.25; Chi² = 25.41, df = 6 (P = 0.0003); I² = 76%								
Test for overall effect: Z = 4.05 (P < 0.0001)								
Test for subgroup differences: Chi² = 3.78, df = 1 (P = 0.05), I² = 73.5%								

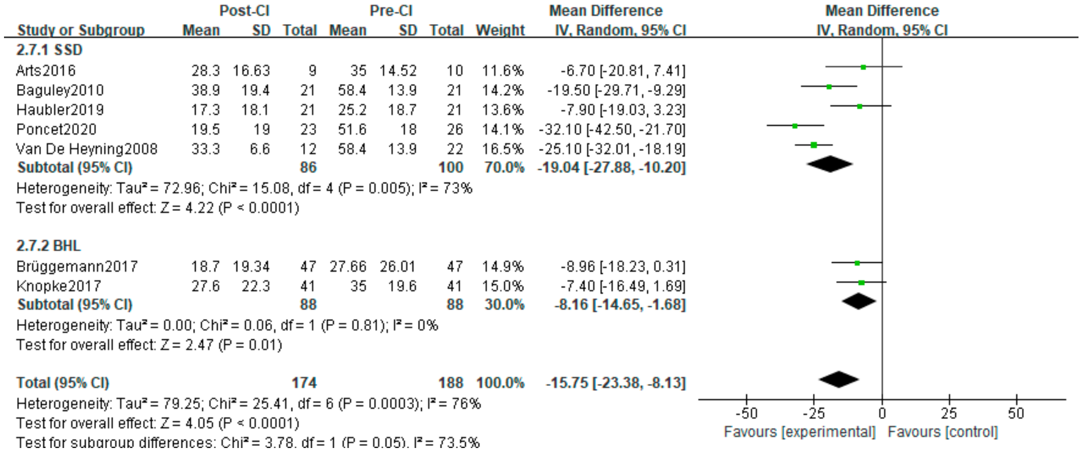

(A)

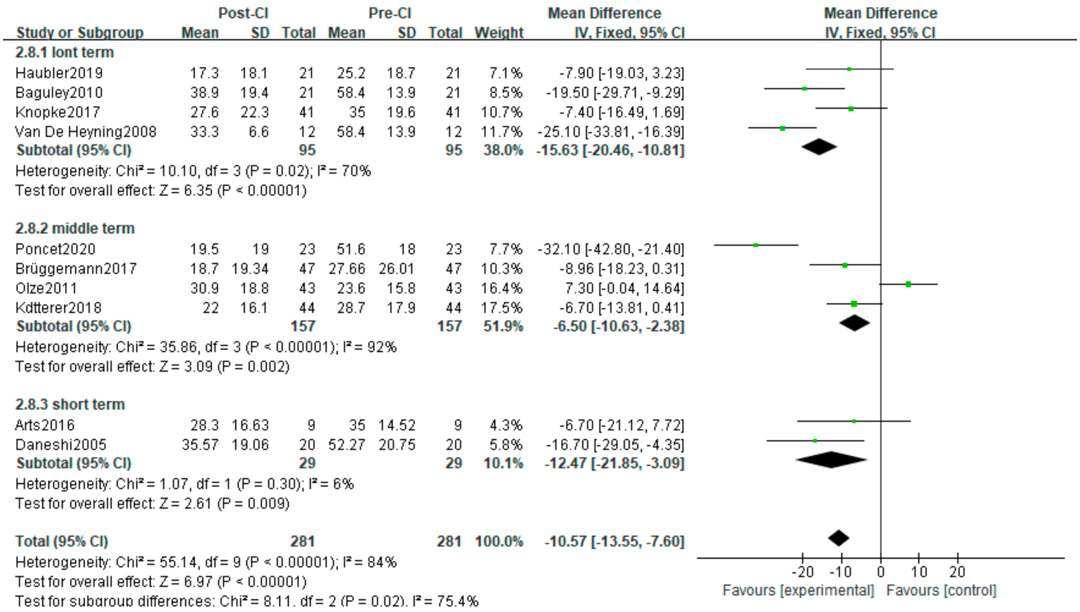

(B)

Figure S3 Outcomes of forest plots from the meta−analysis of VAS: (A) subgroup analysis of single-side-deafness, (B) subgroup analysis for follow-up period, and (C) subgroup analysis for regions. CI, confidence interval

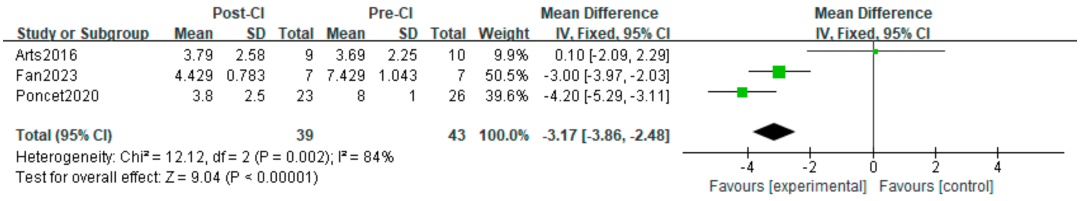

(A)

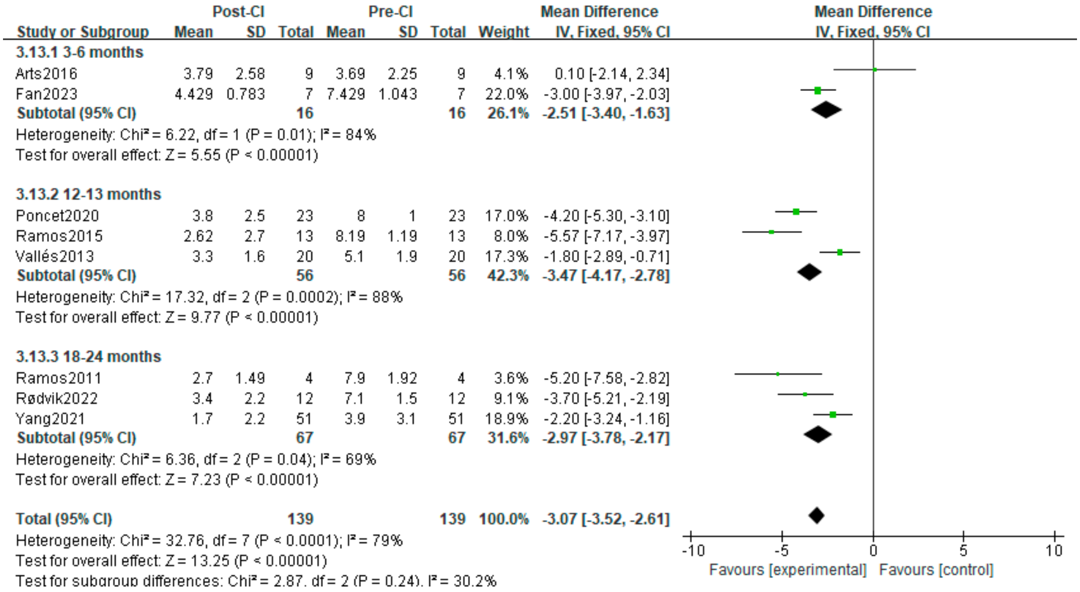

(B)

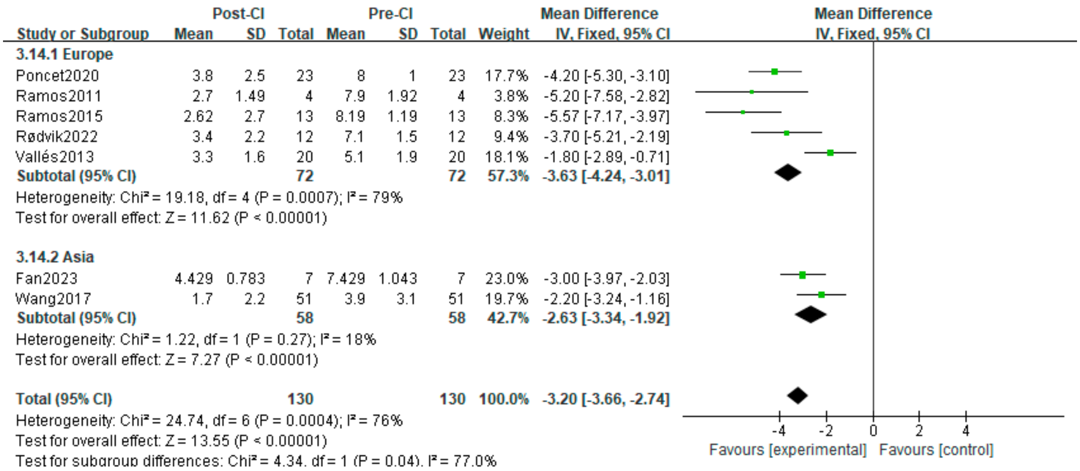
Post-CI
Pre-CI
Mean Difference
Mean Difference
Study or Subgroup Mean SD Total Mean SD Total Weight IV, Fixed, 95% CI
IV, Fixed, 95% CI
3.14.1 Europe
Poncet2020 3.8 2.5 23 8 1 23 17.7% -4.20 [-5.30, -3.10]
Ramos2011 2.7 1.49 4 7.9 1.92 4 3.8% -5.20 [-7.58, -2.82]
Ramos2015 2.62 2.7 13 8.19 1.19 13 8.3% -5.57 [-7.17, -3.97]
Rødvik2022 3.4 2.2 12 7.1 1.5 12 9.4% -3.70 [-5.21, -2.19]
Vallés2013 3.3 1.6 20 5.1 1.9 20 18.1% -1.80 [-2.89, -0.71]
Subtotal (95% CI) 72 72 57.3% -3.63 [-4.24, -3.01]
Heterogeneity: Chi² = 19.18, df = 4 (P = 0.0007); I² = 79%
Test for overall effect: Z = 11.62 (P < 0.00001)
3.14.2 Asia
Fan2023 4.429 0.783 7 7.429 1.043 7 23.0% -3.00 [-3.97, -2.03]
Wang2017 1.7 2.2 51 3.9 3.1 51 19.7% -2.20 [-3.24, -1.16]
Subtotal (95% CI) 58 58 42.7% -2.63 [-3.34, -1.92]
Heterogeneity: Chi² = 1.22, df = 1 (P = 0.27); I² = 18%
Test for overall effect: Z = 7.27 (P < 0.00001)
Total (95% CI) 130 130 100.0% -3.20 [-3.66, -2.74]
Heterogeneity: Chi² = 24.74, df = 6 (P = 0.0004); I² = 76%
Test for overall effect: Z = 13.55 (P < 0.00001)
Test for subgroup differences: Chi² = 4.34, df = 1 (P = 0.04), I² = 77.0%
-4 -2 0 2 4
Favours [experimental] Favours [control]

(C)
